# Coping Strategies and Health-Related Quality of Life in Breast Cancer Survivors

**DOI:** 10.3390/ejihpe15070139

**Published:** 2025-07-17

**Authors:** Ana Agrelo-Fernández, Lucía Fernández-Arce, Ana Llaneza-Folgueras, Ana Isabel Encinas-Muñiz, María Olivo del Valle, Alberto Lana

**Affiliations:** 1 Hospital Universitario San Agustín, Principality of Asturias Health Service, 33401 Aviles, Spain; uo245855@uniovi.es; 2 Department of Preventive Medicine and Public Health, School of Medicine and Health Sciences, University of Oviedo, 33006 Oviedo, Spain; delvalle@uniovi.es (M.O.d.V.); lanaalberto@uniovi.es (A.L.); 3 Instituto de Investigación Sanitaria del Principado de Asturias (ISPA), 33011 Oviedo, Spain; 4 Hospital Universitario Central de Asturias, Principality of Asturias Health Service, 33011 Oviedo, Spain; llanezaana@uniovi.es (A.L.-F.); anaisabel.encinas@sespa.es (A.I.E.-M.)

**Keywords:** breast neoplasms, coping skills, emotions, quality of life

## Abstract

**Background:** The aim was to explore the association between coping strategies (CSs) and health-related quality of life (HRQoL) in breast cancer (BC) survivors and to analyze the role of relevant sociodemographic and clinical variables. **Methods:** A cross-sectional study involving 305 women under follow-up for surgically treated BC in Spain. CSs were measured using the Brief Coping Orientation to Problems Experienced Scale and the HRQoL with the Short-Form Health Survey (SF-12). **Results:** The mean age at BC diagnosis for participants was 57.4 years, with 60.3% of diagnoses at the local stage. Most frequent complementary treatments were radiotherapy (53.4%) and chemotherapy (33.1%). Adaptative CS scores were positively associated both with higher physical HRQoL (adjusted regression coefficient: 2.19; 95% confidence interval: 0.11; 4.27, *p*-value: 0.039) and mental HRQoL scores (coef.: 2.65: 95%CI: 0.25; 5.04, *p*-value: 0.030). Maladaptive CS scores were inversely associated with mental HRQoL scores (coef.: −3.92; 95%CI: −6.62; −1.22, *p*-value: 0.005). The effects were stronger among women with a favorable BC prognosis. **Conclusions:** Adaptive CSs positively affected the physical and mental HRQoL, while maladaptive CSs negatively affected the mental HRQoL. Therefore, psychosocial interventions that promote adaptive CSs and avoid maladaptive ones could improve the well-being of women with a favorable BC prognosis.

## 1. Introduction

Breast cancer (BC) is the most frequently diagnosed malignancy and the leading cause of cancer-related death in most countries (Bray et al., 2024). According to current worldwide estimates from the Global Burden of Disease and GLOBOCAN databases, there are 2.1 to 2.3 million new breast cancer cases and 666,000 to 674,000 breast cancer deaths each year (Deng et al., 2025; Kim et al., 2025). Furthermore, the incidence of BC is expected to continue to rise until 2050 (Deng et al., 2025). In high-income countries, a divergent pattern is observed between the BC incidence and mortality, with slightly increasing incidence rates and decreasing mortality (Giaquinto et al., 2022; Kim et al., 2025), which implies an increase in the population of women who survive BC in the medium to long term. According to the findings of the CONCORD-3 study using data from 322 population-based registries worldwide, the 5-year survival rate for breast cancer increased in virtually all countries for women diagnosed in the period between 2000 and 2014 (Allemani et al., 2018). In Spain, the survival rate was 85.2% in women diagnosed in the period between 2010 and 2014 (Allemani et al., 2018) and is currently estimated to be around 90%.

The diagnosis and treatment of BC is a strong stressor capable of triggering a powerful cascade of physical and mental symptoms and, therefore, of negatively altering the health-related quality of life (HRQoL) (Li & Lambert, 2007; Mehrabi et al., 2015; Compas et al., 2006). Coping strategies (CSs) play a central role in the phase of the adaptation to BC and especially during treatment (Lazarus & Folkman, 2015; Biggs et al., 2017; Mohammadipour & Pidad, 2021). This term refers to the cognitive and behavioral effort made to cope with stressful situations and manage external and internal demands when these exceed personal resources (Lazarus & Folkman, 2015; Biggs et al., 2017).

A growing body of evidence shows that the CSs used by BC survivors are one of the determinants of HRQoL. It is well known that coping with maladaptive CSs, such as avoidance and denial, negatively influences psychological well-being, treatment adherence, and even survival (Mehrabi et al., 2015; Mohammadipour & Pidad, 2021; Reynolds et al., 2000; Watson et al., 2012; Ośmiałowska et al., 2021; Avis et al., 2005). By contrast, dealing with BC using adaptive CSs has a positive impact on all dimensions of HRQoL, as these strategies can mitigate cancer symptoms, decrease the adverse effects of treatment, and improve interpersonal relationships, fostering support (Durosini et al., 2022). In addition, adaptive CSs may promote correct health-related decision making, which, in theory, can improve long-term health outcomes (Durosini et al., 2022).

However, both CSs and HRQoL are dynamic processes, and both can change in response to the problems that the woman is facing at different stages after diagnosis (Torralba-Martínez et al., 2022). In fact, some authors suggest that the association between CSs and HRQoL is indeed bidirectional (Carver et al., 1993; Danhauer et al., 2009). A woman with a low HRQoL because of BC with a worse prognosis or because she is receiving more aggressive treatment may have more difficulty using adaptive CSs. Therefore, it is useful to consider certain clinical variables to understand the association between CSs and HRQoL. Avis et al. conducted a multivariate analysis to find predictors of HRQoL in women with BC younger than 50 years old, including clinical variables related to surgical treatments or symptoms of BC (Avis et al., 2005). According to their results, CSs were stronger predictors of the overall HRQoL than the treatment. However, it is still unknown whether other clinical variables may mediate the association, such as different nonsurgical therapeutic modalities, which are responsible for a growing group of persistent adverse effects (Cathcart-Rake et al., 2023), the time delay, or the stage of the BC. Finally, specifically studying the association between CSs and HRQoL in young, low-educated women may be of interest because they may have more difficulty adjusting to BC than older or college-educated women. Therefore, the aim was to examine the association between CSs and HRQoL in women BC survivors in Spain and to analyze the role of relevant sociodemographic and clinical variables.

## 2. Materials and Methods

### 2.1. Study Design and Participants

This cross-sectional study was conducted in a sample of women under follow-up for BC at the Breast Pathology Unit of the Hospital Universitario Central de Asturias (HUCA). Asturias is a region in northern Spain with about 1 million inhabitants. HUCA is a public, multispecialty, tertiary, and acute care university hospital with 1039 beds. From January to April 2022, participants were recruited consecutively by non-probability sampling using pre-established selection criteria. The inclusion criteria were to have a confirmed diagnosis of BC based on pathological anatomy, to have received surgical treatment for BC, completion of any other primary BC treatment, and no medical diagnosis of cognitive impairment. A woman was considered to have cognitive impairment if her electronic health record showed a medical diagnosis of dementia (ICD-10 codes F00-F03, F05.1, F10.6, G30-G30.9, G31.1, G31.2, G31.8, I67.3) or a score below 24 points on the Mini-Mental State Examination. Women with metastatic or recurrent BC were excluded from the study. Although they are also BC survivors, they were excluded due to their worse clinical prognosis, greater psychosocial challenges, and lack of direct benefits from participating in the study.

**Figure 1:**
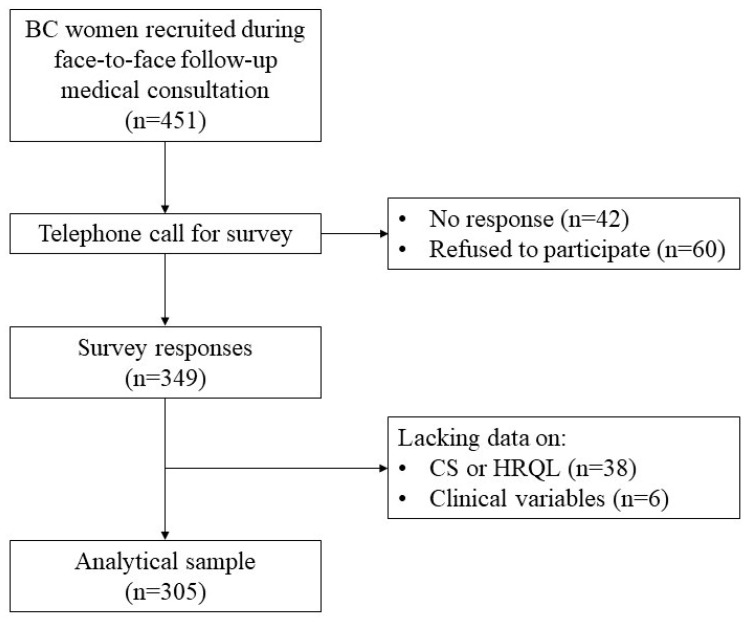
Study flowchart.

According to previous data on the HRQoL of Spanish BC survivors (Alonso-Molero et al., 2020), a sample size of 276 BC women would have 90% power to detect clinically relevant differences in HRQoL scores (2.5 points), considering a 95% confidence interval. During scheduled clinical follow-up visits of BC women, the physicians from the Breast Pathology Unit offered all eligible women the opportunity to participate in this study, for which they provided detailed verbal and written information on the study objectives and procedure. Subsequently, the data collection was carried out by trained nursing staff, who contacted the women to conduct a telephone survey that included the collection of sociodemographic and lifestyle information, and the assessment of CS and HRQoL. Additionally, relevant clinical information was requested from the population-based Tumor Registry of Asturias. Of the 451 women BC survivors who signed the informed consent form, 42 did not answer the telephone after three attempts at three different times, and 60 decided not to participate. Of the 349 completed surveys, 38 were excluded due to missing information on some items used to measure CS and HRQoL. Additionally, 6 women without data on BC treatment were eliminated from the dataset. Thus, the final sample for analysis included 305 BC survivors (Figure 1).

This study was approved by the Ethics Committee for Research in Asturias (ref. 2022.023). All the women provided written informed consent. The decision to participate or not in the study did not affect the women’s relationship with the Breast Pathology Unit. Nor did they receive any type of compensation. This manuscript followed the recommendations of the Strengthening the Reporting of Observational Studies in Epidemiology (STROBE).

### 2.2. Study Variables

#### 2.2.1. Coping Strategies

The CSs were measured using the Brief COPE Scale (Coping Orientation to Problems Experienced) (Carver, 1997), which uses a 4-point Likert scale, ranging from “0: I did not do this at all” to “3: I did this many times”. The Brief COPE measures CSs with 28 items, which are grouped in pairs into 14 factors: active coping, planning, positive reframing, acceptance, humor, religion, emotional support, instrumental support, self-distraction, denial, venting, substance use, behavioral disengagement, and self-incrimination (Rand et al., 2019; Rodrigues et al., 2022). These 14 factors were then grouped into adaptive CSs (the first 8) and maladaptive CSs (the last 6) (Solberg et al., 2022; Dev et al., 2024; Chaaya et al., 2025). Weighted scores (from 0 to 3 points) were calculated by adding the points and dividing by the number of items in each factor. A higher score indicates a greater involvement of the woman with that coping style. The Brief COPE scale has been shown to be reasonably reliable (Rand et al., 2019; Rodrigues et al., 2022). Cronbach’s alpha in our sample was 0.75, indicating good internal consistency.

#### 2.2.2. Health-Related Quality of Life

HRQoL was measured with the Short-Form Health Survey version 2 (SF-12), which assesses the degree of well-being and functional capacity (Kosinski et al., 2007). The SF-12 is composed of 12 items and 8 dimensions, maintaining the conceptual model of the SF-36. The SF-12 scores can be summarized into two global HRQoL indicators, the physical summary component and the mental summary component. The physical and mental HRQoL scores range from 0 to 100 points and are standardized to a national norm with a mean of 50 and a standard deviation of 10; this allows the scores of each study participant to be compared with the mean score of the Spanish population. A higher score on each component indicates a higher HRQoL (Vilagut et al., 2008). Cronbach’s alpha of SF-12 in our sample was 0.88, indicating very good internal consistency.

#### 2.2.3. Other Study Variables

During the telephone survey the women also provided sociodemographic information on cohabitation, number of children, and highest level of education completed. Additionally, the database was completed with relevant clinical information from the Tumor Registry. We considered age at diagnosis, stage of the BC, diagnosis of cancer other than BC (synchronous or prior), diagnostic delay (i.e., days between the suspected diagnosis and confirmation by pathological anatomy), therapeutic delay (i.e., days between definitive diagnosis and initiation of treatment), and other therapeutic modalities complementary to surgery (i.e., chemotherapy, radiotherapy, hormone therapy, targeted therapy and immunotherapy).

#### 2.2.4. Data Analysis

The sociodemographic and clinical characteristics of the study sample were described and mean physical and mental HRQoL scores were calculated according to these variables. Linear regressions were used to study the association between CS scores and physical and mental HRQoL scores. Previously, linear regression assumptions were checked, including normality, linearity, homoscedasticity, independence, and no multicollinearity (Jones et al., 2025). Positive regression coefficients were interpreted as a direct association and negative regression coefficients as an inverse association, with changes representing a unit of change on the scale. Two regression models were conducted to control for the potential effect of different sociodemographic and clinical confounders. In the first model, adjustments were made for age at diagnosis (<45, 45–59, ≥60 years), living arrangements (alone, accompanied), children (none, one, two or more), and level of education (primary, secondary, university). In the second model, we further adjusted for multiple cancers (yes, no), BC stage (local, regional/advanced), diagnostic delay (≤1 week, >1 week), therapeutic delay (≤4 week, >4 week), and treatment modalities (chemotherapy, radiotherapy, hormone therapy, other treatment). In addition, to illustrate a dose–response smoothed association between CS scores and HRQoL, cubic B-splines were used, with automatic determination of the appropriate number and locations of knots.

Also, several sensitivity analyses were performed for a better understanding of the association between CS and HRQoL in strata defined by greater theoretical difficulty in adequately utilizing CS or for CS to materialize in improvements in HRQoL. Thus, linear regression analyses were repeated in women with a diagnosis of BC before the age of 45 years, low educational level, longer total delay time (diagnostic plus therapeutic delay), and more severe BC, i.e., with regional/advanced stage and with higher treatment intensity.

All statistical analyses were performed using STATA v. 18.0 (Stata Corp LLC, College Station, TX, USA). All *p*-values shown were two-tailed, and statistical significance was set at *p* < 0.05.

## 3. Results

Table 1 shows the sociodemographic and clinical characteristics of the study sample. The profile of participants in our study was women aged 45 to 59 years at the time of the BC diagnosis (mean: 57.4; standard deviation: 9.66), with children, living with a companion, and with secondary education. Regarding clinical variables, the most frequent was BC at the local stage and a complementary treatment by radiotherapy. The physical HRQoL was lower in women >60 years, with ≥2 children, with a primary education, and additional treatments. In contrast, the mental HRQoL was lower in women <45 years, with local-stage BC, and no additional treatment.

**Figure 2:**
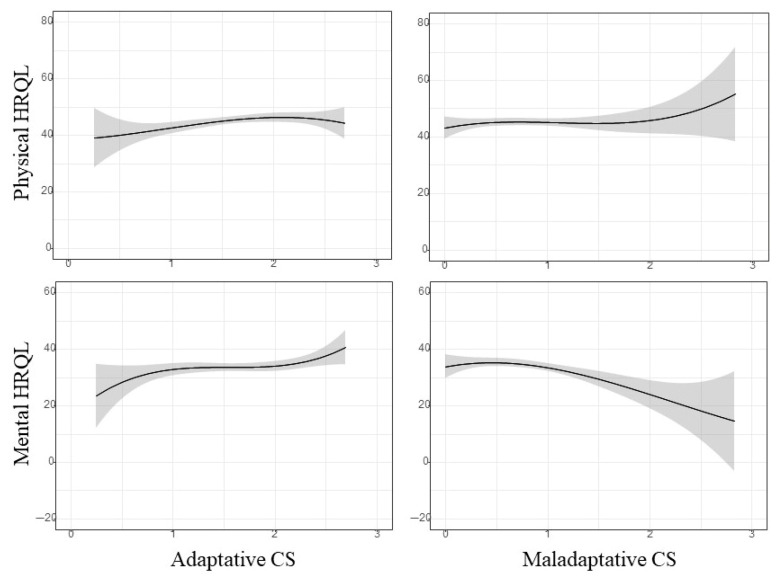
Unadjusted spline curves for the association between adaptative CSs (**top**) and maladaptive CSs (**bottom**) and the physical and mental HRQoL. The solid line represents HRQoL scores based on CS data, and the colored background represents their 95% confidence interval.

Figure 2 represents the unadjusted association between global CS scores and HRQoL scores. A direct and statistically significant association was observed between adaptive CSs and the physical (regression coefficient: 2.63; 95%CI: 0.43; 4.82; *p*-value: 0.019) and mental HRQoL (regression coefficient: 2.91; 95%CI: 0.55; 5.26; *p*-value: 0.016). An inverse association was also found between maladaptive CSs and the mental HRQoL (regression coefficient: −4.51; 95%CI: −7.11; −1.92; *p*-value < 0.001). According to the results in Table 2, these associations are maintained after adjusting for the selected confounders.

Regarding individual CSs, acceptance, active coping, and planning were directly associated with the mental HRQoL, whereas denial, self-blame, and venting were inversely associated with the mental HRQoL (Table 3).

The results of the sensitivity analyses in groups defined by greater vulnerability were consistent in young women, with a lower level of education and a longer delay. However, in women with regional/advanced-stage BC and with ≥2 additional treatments, we found no association between CSs and HRQoL (Table 4).

## 4. Discussion

According to the results of our cross-sectional study in BC survivors in Spain, the use of adaptive CSs was associated with a greater physical and mental HRQOL, and the use of maladaptive CSs was associated with a lower mental HRQOL. The results were independent of the age at diagnosis and educational level; however, CSs were not associated with HRQoL in those with more aggressive BC.

The results of our study are in line with previous research, although the comparison is relatively complex. First, the ambivalent nature of CSs does not facilitate an understanding of their effect on the HRQoL, as the same woman may use adaptive CSs in one situation and maladaptive CSs in another, depending on multiple personal, clinical, and contextual characteristics. Indeed, it is uncommon for a woman BC survivor to feel totally positive or negative throughout the process (Lazarus & Folkman, 2015). Therefore, the timing and context of the measurement may greatly condition the findings. Second, there is a wide variety of conceptualizations of CSs. Thus, the adaptive CSs in our study can be assimilated to active, positive, task-focused, confrontational, or fighting spirit coping; whereas the nomenclature of CSs usually includes destructive, negative, emotional, or resignation coping. Additionally, according to the health theory of coping, all CSs may be adaptive (Stallman, 2020); therefore, they should be labeled as either “healthy” or “unhealthy” based on the risk of adverse consequences.

Regardless of the name used to refer to CSs, most studies worldwide agree with ours that adaptive (healthy) and maladaptive (unhealthy) CSs have antagonistic effects on the HRQoL. The use of adaptive CSs has been directly associated with the HRQoL score, and the use of maladaptive CSs has been inversely associated with it, both in cross-sectional studies (Li & Lambert, 2007; Ośmiałowska et al., 2021; Velasco et al., 2020; Zhou et al., 2022) as well as longitudinal studies (Durá-Ferrandis et al., 2017; Toscano et al., 2020; Senger et al., 2024). Moreover, as in our study, the greatest effect of CSs was detected on the mental component of the HRQoL, probably because clinical factors, such as BC signs, symptoms, treatment, and comorbidities, tend to play a much more important role in physical functioning (Durá-Ferrandis et al., 2017). These findings are plausible with the psychosocial and self-interpretive construct of the HRQoL. A woman with an adaptive approach will be motivated to confront her BC as a challenge that requires personal and social actions to combat it (Mohammadipour & Pidad, 2021; Ośmiałowska et al., 2021). According to the transactional theory (Lazarus & Folkman, 2015; Biggs et al., 2017), adaptive CSs focus on problem solving to change the disturbed person–environment relationship. Focusing on finding solutions reduces stress (Gudenkauf & Ehlers, 2018), which contributes to empowering women in the face of successive challenging situations and consequently to improving their self-concept and HRQoL. Conversely, when using maladaptive CSs, women tend to interpret any symptom as a sign of their declining health due to BC (Mohammadipour & Pidad, 2021; Ośmiałowska et al., 2021) and also become more prone to depressive symptoms (Harel et al., 2025). In addition, the use of maladaptive CSs may compromise information processing during the processing and the expression of emotional states, preventing appropriate psychological adjustments (Toscano et al., 2020; Gutiérrez-Hermoso et al., 2022).

The analysis of the individual CSs that were associated with the mental HRQoL is consistent with the previously stated psychological and behavioral mechanisms. From a general perspective, a CS has a significant impact on HRQoL through the psychological profile and status (Hasan et al., 2024). In our study, women who used acceptance, active coping, and planning achieved higher mental HRQoL scores, probably because accepting the diagnosis of BC without resignation, i.e., having the capacity to fight and to seek tools to manage the initial stress, helps to achieve a good psychological adjustment to the disease (Velasco et al., 2020; Kang et al., 2020). Consistently, Zhou et al. (2022) found that confrontation, which in our study would be a mix of acceptance and active coping, and resignation have opposite effects on HRQoL. While confrontation was associated with better HRQoL scores, resignation was associated with a lower HRQoL (Zhou et al., 2022). In contrast, women who used denial, self-blame, and emotional discharge scored lower on the mental HRQoL. These CSs are not only considered ineffective but are linked to psychological distress. Using these CSs minimizes the inherent capacity of the diagnosis to disrupt daily life and affect the psychological sphere, which may impair the cognitive ability to recognize one’s own emotions, making it difficult to adapt to the stressor (Ben-Zur, 2009). Similarly, Li and Lambert (2007) found that of all the CSs, self-blame was the best predictor of women’s well-being, with a negative contribution to HRQoL in their multiple regression model. Finally, our findings suggest that planning also has a positive individual effect on the physical HRQoL. Women who plan may be better able to anticipate the psychological distress triggered by BC distress, as they spend their time thinking about coping strategies and the steps to take to achieve them. In this process, in addition to accepting distress as an expected human experience, they find solutions to common health problems in BC survivors (e.g., engaging in physical activity, improving diet, attending follow-up visits, etc.).

The major contribution of our study was improving the understanding of the effect of sociodemographic and clinical confounders. This improvement was achieved through the high likelihood that our sample of participants was representative of women treated for BC in Spain due to the nature of the Spanish health system (universal and free), as well as through comprehensive statistical analyses. Many studies have recognized the importance of the clinical characteristics of BC in the association of CSs with HRQoL; however, in practice, they did not perform adequately adjusted analyses (Mohammadipour & Pidad, 2021; Ośmiałowska et al., 2021; Velasco et al., 2020). All our results were consistent even after adjusting for a large number of BC clinical variables, including the stage, delay times, and additional treatments. In addition, our sensitivity analyses in groups of women defined by a greater theoretical difficulty in coping with BC provided highly relevant information. Some previous studies have suggested that younger and less educated women may have more difficulty in employing effective CSs (Kornblith et al., 2007; Calderon et al., 2021; Hernández et al., 2019). However, in our study the association was independent of the age and educational level. The association was dependent on the severity of the BC as estimated by its stage and the number of additional treatments. In women with regional/advanced BC or who required two or more additional treatments, no CSs were associated with HRQoL, suggesting that HRQoL in more aggressive tumors is almost exclusively dependent on clinical issues or that the circumstances surrounding more severe cases exceed the ability of CSs to produce effects. When BC is severe, it is more likely for a woman to experience anxiety, and this makes it difficult to effectively use adaptive CSs (Omari et al., 2022). According to Gutiérrez-Hermoso et al. (2022), when the duration of the treatment is extended, the risk of mental blockage increases, since the treatment sessions demand great effort for emotional processing. In addition, the loss of control that women perceive when they undergo additional treatments with radiotherapy or chemotherapy leads them to visualize a negative prognosis (Gutiérrez-Hermoso et al., 2022; Roszkowska & Białczyk, 2023). Therefore, caution should be exercised with women with more aggressive BC, as expecting them to use adaptive CSs and for these to improve their HRQoL may be an excessive demand, which could trigger a sense of guilt (Velasco et al., 2020; Paek et al., 2016).

Ultimately, in BC cases with a better prognosis, the early identification of individuals at risk of a HRQoL impairment should be followed by tangible clinical and psycho-oncological support, including the promotion of adaptive CSs as an alternative to maladaptive CSs (Durá-Ferrandis et al., 2017). Furthermore, as resilience and a sense of coherence could be underlying causes in the association between CSs and HRQoL, BC surgeons, oncologists, oncology nurses, physiatrists, and psycho-oncologists should also attempt to train these abilities from the outset (Zhou et al., 2022; Zamanian et al., 2021). Lastly, since all individual coping responses may have social counterparts, the family and social environment in which the woman is embedded must also be considered (Skinner et al., 2003). However, in women with BC with a worse prognosis, interventions should be aimed first at mitigating anxiety and the negative symptoms of the disease and treatment, and then we they will be able to work on CSs with a greater guarantee of success. The fear of recurrence is a common and profound concern among advanced BC survivors, plunging women into an anxiety-inducing sea that is difficult to navigate, even using adaptive CSs (Mardani et al., 2025). Community-based interventions organized around workshop sessions and involving both BC survivors and their caregivers have demonstrated improvements in anxiety symptoms. Therefore, these interventions could be considered complementary tools for this initial purpose (Graves et al., 2020). Furthermore, the use of psychosocial support services, whether in-person or via telehealth, has been associated with increases in adaptive CSs and an enhanced HRQoL (Taylor et al., 2025; Walsh et al., 2024). In general, there is a need for multifaceted psychosocial care throughout the oncologic process and to increase health care workers’ knowledge of the coping process. Moreover, since cultural values influence CSs, culturally sensitive interventions should be considered throughout the journey of BC survivors (Hasan et al., 2024).

Our study has some limitations that are important to consider when interpreting the results. First, because of the cross-sectional design we cannot be sure of the direction of the associations. In fact, it has been speculated that the association between CSs and HRQoL is reversible (Danhauer et al., 2009; Paek et al., 2016). Furthermore, CSs and HRQoL are dynamic processes, and therefore the timing of this study may affect the results. An information bias affecting self-reported data cannot be ruled out either, given that the women were recruited from the Breast Pathology Unit and were still in the follow-up phase. Second, we have no information about the type of BC surgery, although it is known that a mastectomy has a greater impact on the psychological outcomes and HRQoL than breast-conserving surgeries (Mishra et al., 2023). Third, our study did not determine the psychological profile of women, and unmeasured psychological variables may underlie both poor coping skills and negative changes in the HRQoL. Fourth, to gain comparability, we decided to use the SF-12, a widely used, generic, and patient-reported measure of the HRQoL. However, more detailed outcomes would probably have been provided by other disease-specific instruments. Finally, the sample size was insufficient for an accurate study of the specific effects of different therapeutic modalities on the association, and participants were recruited from a single hospital. Furthermore, this study excluded women with metastatic BC. This exclusion may have led to an underestimation of the association because women with metastatic BC are expected to exhibit more maladaptive CSs and a worse HRQoL. All these factors may limit the external validity and generalizability of the study results. To bolster our findings, multicenter studies involving samples of BC survivors at different stages of the disease and from various health care settings are necessary.

## 5. Conclusions

In conclusion, our findings provide further evidence for the positive effect of adaptive CSs on the physical and mental HRQoL and for the deleterious effect of maladaptive CSs on the mental HRQoL. The results were independent of the women’s age and educational level but not of the BC severity. The CSs used by women with aggressive BC had no effect on the HRQoL. Our findings add new evidence on the importance of performing interventions in the follow-up consultation of the oncologic process that address emotions and that manage to optimize the use of psychological tools, in order to correctly adjust to the disease in the long term. In the future, it would be interesting to demonstrate the efficacy of these interventions with experimental designs to improve the well-being of BC survivors.

## Figures and Tables

**Table 1:** Sample characteristics and physical and mental HRQoL scores according to sociodemographic and clinical BC variables.

		Total, n (%)	Physical HRQoL, Mean (sd)	Mental HRQoL, Mean (sd)
Participants		305 (100)	45.0 (9.09)	33.9 (9.76)
Age at diagnosis				
	<45 years	80 (26.2)	46.0 (9.91)	32.7 (8.33)
	45–59 years	160 (52.5)	46.0 (8.47)	33.5 (9.92)
	≥60 years	65 (21.3)	41.4 (9.99)	36.0 (10.7)
Living arrangement				
	Lives alone	54 (17.7)	44.6 (9.22)	33.8 (10.5)
	Accompanied	251 (82.3)	45.1 (9.08)	33.9 (9.61)
Children				
	None	69 (22.6)	48.1 (6.27)	33.8 (8.52)
	1 child	91 (29.8)	46.4 (8.29)	33.8 (9.84)
	2 or more children	145 (47.6)	42.7 (10.1)	33.9 (10.3)
Level of studies				
	Primary	47 (15.4)	39.5 (11.1)	33.5 (9.96)
	Secondary	168 (55.1)	44.5 (8.68)	33.5 (10.1)
	University	90 (29.5)	49.0 (6.71)	34.7 (8.93)
Multiple cancers				
	No	266 (87.2)	45.1 (9.02)	34.1 (9.42)
	Yes	39 (12.8)	44.3 (9.69)	32.5 (11.9)
Stage of BC				
	Local	184 (60.3)	44.9 (9.40)	32.3 (9.97)
	Regional or advanced	121 (39.7)	45.1 (8.64)	36.2 (8.96)
Diagnostic delay ^a^				
	≤1 week	148 (48.5)	46.1 (8.25)	34.3 (9.72)
	>1 week	157 (51.5)	44.0 (9.75)	33.4 (9.80)
Therapeutic delay ^a^				
	≤4 weeks	149 (48.9)	44.7 (9.19)	34.1 (9.67)
	>4 weeks	156 (51.1)	45.3 (9.02)	33.6 (9.86)
Chemotherapy				
	No	204 (66.9)	45.1 (9.21)	33.1 (9.70)
	Yes	101 (33.1)	44.8 (8.89)	35.3 (9.75)
Radiotherapy				
	No	142 (46.6)	46.1 (9.01)	33.0 (9.88)
	Yes	163 (53.4)	44.0 (9.08)	34.6 (9.62)
Hormone therapy				
	No	207 (67.9)	45.9 (9.03)	33.9 (9.92)
	Yes	98 (32.1)	43.1 (8.98)	33.8 (9.44)
Targeted/Immunotherapy				
	No	289 (94.7)	45.0 (9.14)	33.8 (9.79)
	Yes	16 (5.25)	45.8 (8.37)	34.9 (9.41)

HRQoL, health-related quality of life; BC, breast cancer; and SD, standard deviation. ^a^ Cut-off point: median time (weeks).

**Table 2:** Regression coefficients (95%CI) for the association between grouped coping strategies scores and physical and mental HRQoL scores.

		Physical HRQoL		Mental HRQoL	
		Coefficient (95%CI)	*p*-Value	Coefficient (95%CI)	*p*-Value
Adaptive CS					
	Model 1 ^a^	2.10 (0.04; 4.16)	0.046	2.77 (0.37; 5.16)	0.024
	Model 2 ^b^	2.19 (0.11; 4.27)	0.039	2.65 (0.25; 5.04)	0.030
Maladaptive CS					
	Model 1 ^a^	0.10 (−2.26; 2.46)	0.931	−3.97 (−6.69; −1.27)	0.004
	Model 2 ^b^	0.02 (−2.35; 2.40)	0.984	−3.92 (−6.62; −1.22)	0.005

CSs: coping strategies; CI, confidence interval; HRQoL: health-related quality of life. ^a^ Model 1 adjusted for age at BC diagnosis (<45, 45–59, ≥60 years), living arrangements (alone, accompanied), children (none, one, two or more) and educational level (primary, secondary, university). ^b^ Model 2 further adjusted for multiple cancer (yes, no), BC stage (local, regional/advanced), diagnostic delay (≤1 week, >1 week), therapeutic delay (≤4 week, >4 week) and treatment modalities (chemotherapy, radiotherapy, hormone therapy, targeted therapy and immunotherapy).

**Table 3:** Regression coefficients (95%CI) for the association between individual CS scores and physical and mental HRQoL scores.

		Physical HRQoL		Mental HRQoL	
		Coefficient (95%CI) ^a^	*p*-Value	Coefficient (95%CI) ^a^	*p*-Value
Adaptive CSs					
	Emotional support	0.04 (−1.11; 1.19)	0.941	−0.10 (−1.43; 1.22)	0.877
	Positive reframing	0.73 (−0.34; 1.80)	0.182	0.63 (−0.56; 1.91)	0.280
	Acceptance	0.93 (−0.55; 2.40)	0.217	2.78 (1.11; 4.46)	0.001
	Religion	0.55 (−0.36; 1.47)	0.235	0.28 (−077; 1.34)	0.598
	Humor	1.01 (−0.19; 2.21)	0.100	0.52 (−0.87; 1.91)	0.465
	Active coping	0.84 (−0.31; 1.99)	0.153	2.26 (0.95; 3.57)	0.001
	Planning	1.11 (0.00; 2.21)	0.050	1.43 (0.16; 2.71)	0.027
	Instrumental support	0.04 (−1.01; 1.10)	0.937	−0.55 (−1.77; 0.66)	0.371
Maladaptive CSs					
	Denial	−0.90 (−1.90; 0.09)	0.075	−1.91 (−3.05; −0.78)	0.001
	Substance use	0.70 (−2.12; 3.53)	0.624	−1.73 (−4.98; 1.51)	0.293
	Behavioral disengagement	−0.81 (−2.47; 0.84)	0.334	−1.81 (−3.71; 0.08)	0.060
	Self-distraction	0.83 (−0.15; 1.82)	0.099	0.76 (−0.37; 1.90)	0.188
	Self-blame	0.09 (−1.59; 1.78)	0.911	−2.41 (−4.33; −0.49)	0.014
	Venting	0.34 (−0.81; 1.50)	0.554	−1.73 (−3.04; −0.42)	0.010

CSs: coping strategies; CI, confidence interval; and HRQoL: health-related quality of life. ^a^ Linear regression adjusted for age at BC diagnosis (<45, 45–59, ≥60 years), living arrangements (alone, accompanied), children (none, one, two or more), educational level (primary, secondary, university), multiple cancers (yes, no), stage of BC (local, regional/advanced), diagnostic delay (≤1 week, >1 week), therapeutic delay (≤4 week, >4 week), and treatment modalities (chemotherapy, radiotherapy, hormone therapy, targeted therapy, and immunotherapy).

**Table 4:** Regression coefficients (95%CI) for the association between pooled CS scores and physical and mental HRQoL scores. Sensitivity analysis.

		Physical HRQoL		Mental HRQoL	
		Coefficient (95%CI) ^a^	*p*-Value	Coefficient (95%CI) ^a^	*p*-Value
Adaptive CSs					
	<45 years old	2.98 (0.40; 5.56)	0.024	2.99 (−0.11; 6.10)	0.059
	High school or less	2.79 (0.15; 5.43)	0.038	2.85 (−0.03; 5.74)	0.053
	Total delay ^b^ ≥6 weeks	4.27 (0.96; 7.58)	0.012	0.92 (−3.08; 4.92)	0.649
	Regional/advanced stage	−1.86 (−4.99; 1.26)	0.240	0.61 (−2.90; 4.13)	0.730
	≥2 additional treatments	1.46 (−2.11; 5.02)	0.420	1.51 (−2.71; 5.73)	0.480
Maladaptive CSs					
	<45 years old	−1.07 (−3.98; 1.82)	0.465	−4.66 (−8.09; −1.24)	0.008
	High school or less	1.32 (−1.60; 4.25)	0.373	−3.80 (−6.96; −0.64)	0.019
	Total delay ^b^ ≥6 weeks	1.52 (−1.87; 4.92)	0.376	−4.68 (−8.61; −0.76)	0.020
	Regional/advanced stage	−0.92 (−4.64; 2.78)	0.621	0.13 (−4.03; 4.29)	0.952
	≥2 additional treatments	−3.22 (−7.24; 0.80)	0.115	−3.11 (−7.88; 1.66)	0.199

CSs: coping strategies; CI, confidence interval; and HRQoL: health-related quality of life. ^a^ Linear regression adjusted for age at BC diagnosis (<45, 45–59, ≥60 years), living arrangements (alone, accompanied), children (none, one, two or more), educational level (primary, secondary, university), multiple cancer (yes, no), stage of BC (local, regional/advanced), diagnostic delay (≤1 week, >1 week), therapeutic delay (≤4 week, >4 week), and treatment modalities (chemotherapy, radiotherapy, hormone therapy, targeted therapy, and immunotherapy). ^b^ Total delay = diagnostic plus therapeutic delay.

## Data Availability

Research data are available on request from the authors.

## Abbreviations

The following abbreviations are used in this manuscript:

BC	Breast cancer
CSs	Coping strategies
HRQoL	Health-related quality of life
STROBE	Strengthening the Reporting of Observational Studies in Epidemiology
